# Supplementary material for: Rapid turnover of life-cycle-related genes in the brown algae
Source: Genome Biol. 2019 Feb 14;20:35. doi: 10.1186/s13059-019-1630-6 (PMC6374913; doi:10.1186/s13059-019-1630-6)
Supplement: Supplementary file 3 — Protein alignments, corresponding Gblocks html file and CDS sequences for the lineage conserved gametophyte-biased genes. (ZIP 2109 kb) [file 13059_2019_1630_MOESM3_ESM.zip › Con_GA_alignments/Ec-07_004200.aa_ali.fasta-gb.htm]

Ec-07\_004200.aa.fasta.trans.aa\_ali.fasta


## Gblocks 0.91b Results

Processed file: **Ec-07\_004200.aa.fasta.trans.aa\_ali.fasta**  
Number of sequences: **2**  
Alignment assumed to be: **Protein**  
New number of positions: **440** (selected positions are underlined in blue)

```
                         10        20        30        40        50        60
                 =========+=========+=========+=========+=========+=========+
Ec-07_004200     MQSALMAGGLTPALRQLKDCRHTFPSSKWDTRDSQGAPRHGQGCLTCNGPRPERPTELEP
MSTRG.44255.1    -----------------PERAENAESEDATAADAIDVDSAGVRDVSDYDTAVERKPEHHQ
                                          ################           ########


                         70        80        90       100       110       120
                 =========+=========+=========+=========+=========+=========+
Ec-07_004200     NNETGTGDFGAILNGIEEGGRVERCGGDSRPKLVPGAMLTFSNTAVAAVKAFAEEVLAEE
MSTRG.44255.1    HNSR-----AQATEHVRPGDRVGGNVGDNRPKLAPGAMLTLSHLATEAMMAFTQEVLDEE
                 ##                ##########################################


                        130       140       150       160       170       180
                 =========+=========+=========+=========+=========+=========+
Ec-07_004200     DPPRRATKAAAIVKEATTEVSFANRRNHPRTEVLVTRLQAVVRGWLARRLARVLLLRGGC
MSTRG.44255.1    DPKERAKKAVAIVTEATSEVAFATRRGRPRTGVLVTKLQAVARGWLARRQARVLVLRGGC
                 ############################################################


                        190       200       210       220       230       240
                 =========+=========+=========+=========+=========+=========+
Ec-07_004200     RRELDVASGAWQYVWSHRAAVPGAPGAAATAPGRASYAGGIGGGGYRSPACGGDGGEGLF
MSTRG.44255.1    RRELDIASGAWQYVWSFRAGVP-----DPVVDKRRGYS---VSPSPPPAGNGGGGGPGLF
                 ######################                             #########


                        250       260       270       280       290       300
                 =========+=========+=========+=========+=========+=========+
Ec-07_004200     RTWYPPALLKNESLPSPRAAARRVEGEGRKREARLALANSLLDDQRAELNRRDARAARLL
MSTRG.44255.1    RSWYPPALLKGETLPSPRAAVRRVEGEERKREARLALANSLLDGQRAQLERRQARAVRLL
                 ############################################################


                        310       320       330       340       350       360
                 =========+=========+=========+=========+=========+=========+
Ec-07_004200     ALVEVVKLVERAMASLEEAALTCFGTGSEPLAISVKVLHPRGGEPGDGAREAVVTEIMSA
MSTRG.44255.1    ALAEVVKLVERATVNLEGAALGCFGISSKPLCISLRVFHPRGGQPGDGAREAVVSEIMAA
                 ############################################################


                        370       380       390       400       410       420
                 =========+=========+=========+=========+=========+=========+
Ec-07_004200     WHRIDAQVREQEEAARLRAERTNDKGKSPKT-SPNRGGGVSARRTPRSGSREGRGKSTGR
MSTRG.44255.1    WRRVDTQVREQKEAARIRAERAGGKGRTQRLNSPSRGTGGGGKRTPRSTSPERRGK----
                 ##########################      ########################    


                        430       440       450       460       470       480
                 =========+=========+=========+=========+=========+=========+
Ec-07_004200     REAKLSVPTTNENLAAFEQ-------------IAARLLARRRVR-----GG--GLSSPEE
MSTRG.44255.1    ---KLAVPTTNDNLAEFEKRLLQDSLHEDASAAVASLLQRKRVLVAIHEASRLGRNKDAW
                    ###############                                   #######


                        490       500       510       520       530       540
                 =========+=========+=========+=========+=========+=========+
Ec-07_004200     ASARLHPRGFKIQVFPPWALASHRFIALVHRGSLVAVSQASPFVFWENLADRKKTNQLIA
MSTRG.44255.1    EKAGTSPFGLGVQVLPPWARPSHRFIALAHRGTLVAVSQASPFVFWETLADRETLSRVLR
                 ####################################################        


                        550       560       570       580       590       600
                 =========+=========+=========+=========+=========+=========+
Ec-07_004200     DILGPFLENPDLLKAAFADEPGERASVPATKATSAGRSTTNNAVSTARETPGDGAGGDGE
MSTRG.44255.1    EILGPFLDNADLLKGMFVG------GQLSNPASAAGSTTTN-------------------
                  ################              ##########                   


                        610       620       630       640       650       660
                 =========+=========+=========+=========+=========+=========+
Ec-07_004200     QSTSSSVAEAGDTGVRQIGRENEEGSAAAAAAAAADGGMWGYRSSLSSKGCWKIDARAVA
MSTRG.44255.1    --------------------------AHRFTASAADGX----------------------
                                           ###########                       


                        670       680       690       700       710       720
                 =========+=========+=========+=========+=========+=========+
Ec-07_004200     RRDSSLKLGFDSVAFDLFVLPDEKKGGKKGGQEPPAAPSVGKQRHATRVNDHEEDESSSP
MSTRG.44255.1    ------------------------------------------------------------
                                                                             


                        730       740       750       760       770       780
                 =========+=========+=========+=========+=========+=========+
Ec-07_004200     RATRAVVTNAVPFRLADGLPRELGPLLFEDTSDASLLAGDAAEDHPQSRGAKRKPEHPRV
MSTRG.44255.1    ------------------------------------------------------------
                                                                             


                        790       800       810       820       830       840
                 =========+=========+=========+=========+=========+=========+
Ec-07_004200     TVSDPELVGSRPEEAEEETEEEEGYEWERKKDPRSAGKILSHTRRGSGPSEAAVEVRIRG
MSTRG.44255.1    ------------------------------------------------------------
                                                                             


                        850       860       870       880       890
                 =========+=========+=========+=========+=========+
Ec-07_004200     ETLGADQLARAGLPHDLFSLVVDERFKVTNAATAGDSKSTRGRTKGRRKE
MSTRG.44255.1    --------------------------------------------------
```

```
Parameters used
Minimum Number Of Sequences For A Conserved Position: 2
Minimum Number Of Sequences For A Flanking Position: 2
Maximum Number Of Contiguous Nonconserved Positions: 8
Minimum Length Of A Block: 10
Allowed Gap Positions: None
Use Similarity Matrices: Yes
```

```
Flank positions of the 10 selected block(s)
Flanks: [26  41]  [53  62]  [79  202]  [232  386]  [393  416]  [424  438]  [474  532]  [542  557]  [572  581]  [627  637]  

New number of positions in Ec-07_004200.aa.fasta.trans.aa_ali.fasta-gb:  440  (49% of the original 890 positions)
```
